# Mucin pattern reflects the origin of the adenocarcinoma in Barrett's esophagus: a retrospective clinical and laboratorial study

**DOI:** 10.1186/1477-7819-7-27

**Published:** 2009-03-09

**Authors:** Sergio Szachnowicz, Ivan Cecconello, Ulysses Ribeiro, Kiyoshi Iriya, Roberto El Ibrahim, Flávio Roberto Takeda, Carlos Eduardo Pereira Corbett, Adriana Vaz Safatle-Ribeiro

**Affiliations:** 1 Department of Gastroenterology, Digestive Surgery Division, University of São Paulo School of Medicine, São Paulo, Brazil; 2 Department of Pathology, University of São Paulo School of Medicine, São Paulo, Brazil

## Abstract

**Background:**

Mucin immunoexpression in adenocarcinoma arising in Barrett's esophagus (BE) may indicate the carcinogenesis pathway. The aim of this study was to evaluate resected specimens of adenocarcinoma in BE for the pattern of mucins and to correlate to the histologic classification.

**Methods:**

Specimens were retrospectively collected from thirteen patients who underwent esophageal resection due to adenocarcinoma in BE. Sections were scored for the grade of intestinal metaplasia. The tissues were examined by immunohistochemistry for MUC2 and MUC5AC antibodies.

**Results:**

Eleven patients were men. The mean age was 61 years old (varied from 40 to 75 years old). The tumor size had a mean of 4.7 ± 2.3 cm, and the extension of BE had a mean of 7.7 ± 1.5 cm. Specialized epithelium with intestinal metaplasia was present in all adjacent mucosas. Immunohistochemistry for MUC2 showed immunoreactivity in goblet cells, while MUC5AC was extensively expressed in the columnar gastric cells, localizing to the surface epithelium and extending to a variable degree into the glandular structures in BE. Tumors were classified according to the mucins in gastric type in 7/13 (MUC5AC positive) and intestinal type in 4/13 (MUC2 positive). Two tumors did not express MUC2 or MUC5AC proteins. The pattern of mucin predominantly expressed in the adjacent epithelium was associated to the mucin expression profile in the tumors, p = 0.047.

**Conclusion:**

Barrett's esophagus adenocarcinoma shows either gastric or intestinal type pattern of mucin expression. The two types of tumors developed in Barrett's esophagus may reflect the original cell type involved in the malignant transformation.

## Background

Barrett's esophagus (BE) is the eponymous term used to describe a condition with malignant potential where the lower esophagus becomes lined with a specialized columnar epithelium as a result of chronic gastroesophageal reflux. Nowadays, Barrett's esophagus represents the transition from normal squamous mucosa to columnar epithelium plus the identification of intestinal metaplasia. In macroscopic form, BE is classified as long, when the columnar epithelium is longer than 3 cm, and short when is lower than 3 cm [1,2].

BE is a complex, mosaic of cell, gland, and architectural types, showing variable degrees of atrophy and maturation toward intestinal and gastric epithelium. Surface mucous, goblet cells, absorptive, mucous neck, mucous gland and neuroendocrine cells are randomly distributed in relation to the gastroesophageal junction [3,4].

Although there are three types of columnar epithelium – namely, gastric fundic, junctional cardiac and specialized intestinal epithelium, – it is now accepted that adenocarcinoma arises only from the specialized intestinal-type of metaplasia [3,5-12]. Nonetheless, many of the esophageal adenocarcinomas in BE (ABE) exhibit a poor differentiated and/or undifferentiated pattern, distinct from the intestinal type tumors commonly observed in patients with intestinal metaplasia.

Mucin genes are expressed throughout the human gastrointestinal tract in a site specific manner [13]. In specialized BE, there is strong expression of MUC2 in the goblet cells (intestinal mucin pattern) and MUC5AC in the superficial columnar epithelium (gastric mucin pattern) [14]. This is the same pattern already described for incomplete intestinal metaplasia of the stomach, and is further evidence that BE and incomplete intestinal metaplasia of the stomach are the same condition and represent differentiation into a unique epithelial lineage [15,16].

BE is a marker of tissue injury possibly as a consequence of inflammatory lesions and regeneration. Thus, all cells of the BE under damage could originate an expansion clone capable of initiate the carcinogenesis cascade. The pattern of expression of mucin gene products in adenocarcinoma arising in BE has yet to be known.

Thus, we have studied a homogenous group of patients with adenocarcinoma arising in BE. We sought to determine whether gastric (MUC5AC) and/or intestinal type (MUC2) markers, could help improve our understanding of the carcinogenesis in Barrett's adenocarcinoma.

## Patients and methods

This investigation was approved by the Ethical Committee of the Hospital das Clínicas of São Paulo Medical School. From January, 1990 to June, 2002, a total of 297 patients with diagnostic of BE confirmed through endoscopic biopsies, were treated at the Esophageal Surgery Service of Digestive Surgery Division of Hospital das Clínicas of the University of São Paulo School of Medicine. Of those, Adenocarcinoma was diagnosed in 17 patients, with a prevalence of 5.7%. We retrospectively review the clinical charts of these patients regarding the presence of Barrett's esophagus, clinical characteristics and pathology report. Gastric tumors with esophageal invasion and esophageal neoplasias with invasive components to the gastric cardia were excluded. Carcinomas were deemed to be arising from the Barrett's esophagus, if, on histological examination, there was specialized columnar metaplasia proximal and/or involving the tumor.

Among the 17 patients, three were excluded due to unresectable advanced neoplasia. One underwent argon plasmatic ablation of the columnar epithelium, including the tumor, which was not identified in the histopathologic study of the resected esophagus. The remaining 13 patients underwent esophageal resection, and form the basis of this study.

### Histopathologic study

All the pathological specimens are prepared according to the Pathology Department guidelines. The resected esophagus was opened longitudinally, photographed, stretched in glides of plastic or cardboard surface, BE and tumor extension were measured. The distances between tumor's distal margins and gastroesophageal junction (Dist. Tu-GEJ); and tumor's proximal margins and columnar-squamous transition (Prox. Tu-Tepit) were performed. After this, the specimens were fixed using formaldehyde solution.

For the histological study, tissue samples were retrieved from archived paraffin embedded sections of histologically known Barrett's esophagus. Tumor and adjacent epithelium, were stained by hematoxyline-eosine (HE).

Histology of the adjacent tumor area showed a specialized-type mucosa characterized by an epithelial lining which included columnar epithelium showing a poorly developed brush border, villous architecture, and goblet cells. The surface cells were of surface mucous type, with underlying cardiac/antral glands beneath surfaces covered by goblet and absorptive cells. Barrett's esophagus could be classified as specialized epithelium in all studied patients, with areas with predominance of an intestinal or gastric type epithelium in each patient.

The tumors were classified according to the grade of differentiation.

### Immunohistochemical evaluation

Sections of tumors, and corresponding adjacent areas, developing in patients with Barrett's esophagus were examined by immunohistochemistry for MUC5AC (NCL – MUC-5AC, Novocastra, Newcastlle, United Kingdom) and MUC2 (NCL – MUC-2, Novocastra, Newcastlle, United Kingdom).

Three to five unstained 4 μm blank histologic sections were cut from each designated block and used for MUCAC-5 and MUC-2 immunostaining (using humid heating). Briefly, immunodetection involved the use of 4 μm thick formalin-fixed paraffin-embedded tissues, treated with 4% and 2% hydrogen peroxidase (H_2_O_2_) in methanol for 35 minutes, to eliminate endogenous peroxidase activity. Sections were rinsed in phosphate-buffered saline (PBS) and incubated with 10% normal horse serum to block nonspecific binding. Upon removal of the serum, the primary monoclonal antibody was applied. Following further washing with PBS, sections were incubated with biotinylated anti-mouse immunoglobulin for 30 minutes. After washing twice with PBS, the sections were treated with Vectastain Elite horseradish peroxidase complex (Vector Laboratory, Burlingame, CA) for 30 minutes. Following another rinse with PBS, the sections were incubated with diaminobenzidine 0.05% and 0.04% H_2_O_2 _for 20 minutes. After a final wash with distilled water, the sections were counterstained with Harris Alum Hematoxylin, dehydrated through graded alcohols to xylene, and coverslipped.

All sections were examined by three independent investigators (KY, REI and UR) for the histopathological study and blindly for immunohistochemical evaluation by the third one. The mucins were expressed as cytoplasmic staining. The results were expressed semiquantitatively for each histological group as the number of sections positively labeled, the predominant cell type labeled, and the average score of the positively labeled cells. Positive Control Sections: control tissues taken from colon and stomach, with previously identified MUC gene expression patterns were included with each batch of sections for immunohistochemistry.

Negative Control Sections: the primary antibody was omitted as a negative control to test the specificity of the antibodies utilized for each section.

Incubation with Primary Antibody (MUC2 was diluted in 1:100, and the MUC5, 1:400)

### Statistical analysis

Results of immunohistochemical alterations were compared to the clinical-pathologic features using chi-square test for qualitative data, with two tailed p value < 0.05 considered significant.

## Results

Eleven patients were men (84.6%) and two women (15.4%), with proportion of 5.5:1. The age range from 40 to 75 years-old (mean = 61 years ± 9.9).

### Histopathological results

Measurements obtained from each resected esophagus are presented in table 1. Columnar epithelium extension ranged from 3.5 to 16.0 cm (mean of 7.7 ± 3.3 cm). Tumor extension ranged from 1.5 to 7.4 cm (mean of 4.7 ± 2.3 cm). All adenocarcinoma developed in BE longer than 3.0 cm. The distances between the tumor's distal margins and gastroesophageal junction (Dist. Tu-GEJ) ranged from just at the GEJ (5 patients – 38.5%) to tumors 14 cm far from GEJ (mean of 2.1 cm). The distances of tumor's proximal margins and columnar-squamous transition (Prox. Tu-Tepit) ranged from tumors that reached the epithelium transition and tumor 3.5 cm far from Tepit (mean of 1.30 cm). Eight tumors (61.5%) were located less than 1.0 cm of the columnar-squamous transition.

**Table 1:** Lengths of barrett's esophagus epithelium and adenocarcinoma.

Patient	Barrett's esophagus length (cm)	Adenocarcinoma length (cm)	Dist. Tu-GEJ (cm)	Prox. Tu-Tepit (cm)
1	16	3.6	14	0.4
2	10	8	0.5	1.5
3	4	3.0	1	0
4	7	6.5	0	0.5
5	8	5	0	3
6	6	7.4	2.2	0
7	3.5	3	0	0.5
8	5	4.5	0.3	0.5
9	10.7	2.2	5.5	2.5
10	8	7	0	1
11	6.5	1.5	1.5	3.5
12	9.5	7	2.5	0
13	6	2.5	0	3.5
Mean (SD)	7.71 (3.33)	4.67 (2.28)	2.07	1.30
Min	3.5	1.5	0	0
Max	16	7.4	14	3.5

Distances from adenocarcinoma to gastroesophageal junction; distances from adenocarcinoma to squamous-columnar transition.

Dist. Tu-GEJ = Distance from tumors (Adenocarcinoma) distal margin to the gastroesophageal junction.

Prox. Tu-Tepit = Distance from the turmors (Adenocarcinoma) proximal margin to the epithelium (columnar-squamous) transition

Histopathological classifications of adenocarcinomas and their adjacent columnar epithelium are presented in table 2. Four tumors were well differentiated, two moderated, five were poorly and two were undifferentiated. The adjacent epithelium was specialized columnar type. In five cases there was predominance of intestinal type areas; five, with predominance of gastric type areas, and three with similar distribution.

**Table 2:** Distribution of 13 ABE patients according to the type of adjacent epithelium and tumor

Characteristics					
**Patient**	**Cell type (gastric or intestinal) predominance in the specialized columnar epithelium**		**Adenocarcinoma**		

		Grade of	IHC	IHC	Type of tumor
		differentiation	MUC2	MUC5AC	according to mucins
1	intestinal	well	+	-	Intestinal
2	intestinal	moderated	-	+	Gastric
3	similar	well	-	+	Gastric
4	intestinal	moderated	+	-	Intestinal
5	Gastric	poor	-	+	Gastric
6	Gastric	undifferentiated	-	-	-
7	Gastric	well	-	+	Gastric
8	Intestinal	poor	+	-	Intestinal
9	Gastric	poor	-	+	Gastric
10	similar	well	-	+	Gastric
11	similar	undifferentiated	-	-	-
12	Gastric	poor	-	+	Gastric
13	Intestinal	poor	+	-	Intestinal

IHC = immunohistochemistry

### Immunohistochemical results

**Figure 1:**
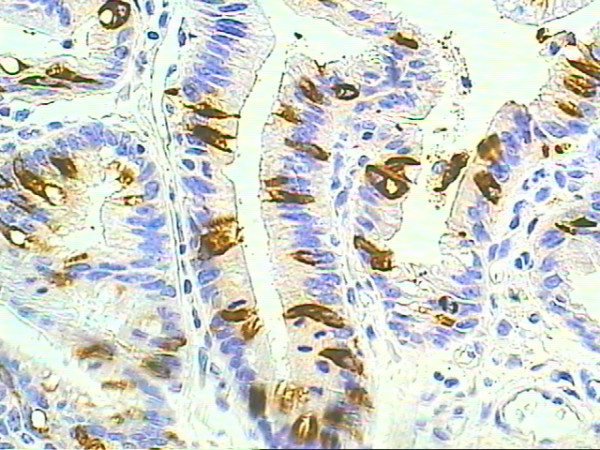
**MUC2 immunoexpression in columnar epithelium adjacent to the Adenocarcinoma**. Immunohistochemical staining of MUC2 for columnar epithelium showing goblet cells as positive control (original magnification × 400)

**Figure 2:**
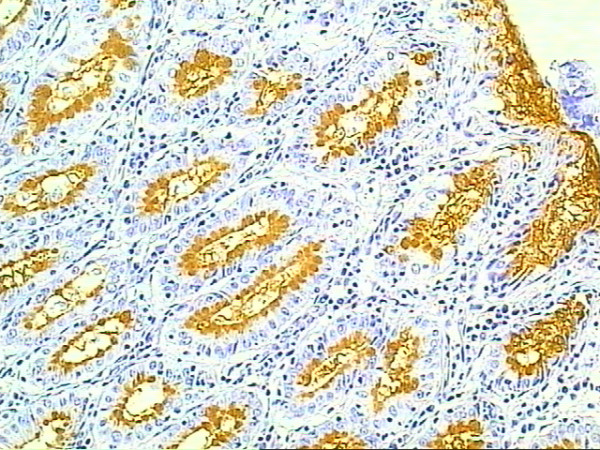
**MUC5AC immunoexpression in columnar epithelium adjacent to the Adenocarcinoma**. Immunohistochemical staining of MUC5AC for columnar epithelium showing glandular structures as positive control (original magnification × 400)

Immunohistochemical analysis of mucins is presented in table 2. Normal esophagus epithelium was usually seen in the sections, often continuous with the BE epithelium. The mucins were not expressed in the esophageal normal stratified epithelium. Intestinal metaplasia with goblet cells was usually found at the mucosal surface, and in some cases it was seldom detected. MUC2 was associated specifically with goblet cells in IM and was usually found at the mucosal surface (Figure 1). Patches of IM within BE were characterized by expression of MUC2 within goblet cells, which is also characteristic for normal intestinal epithelium and for IM in stomach. MUC5AC was extensively expressed in BE columnar epithelium, localizing to the surface epithelium and extending to a variable degree into the glandular structures (Figure 2). No MUC5AC staining was detected in goblet cells.

**Figure 3:**
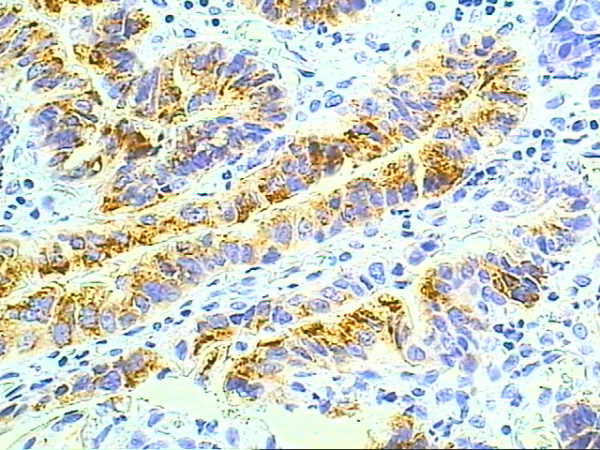
**MUC2 immunoexpression in intestinal type adenocarcinoma arising in Barrett's esophagus**. Immunohistochemical staining of MUC2 for adenocarcinoma (original magnification × 400)

**Figure 4:**
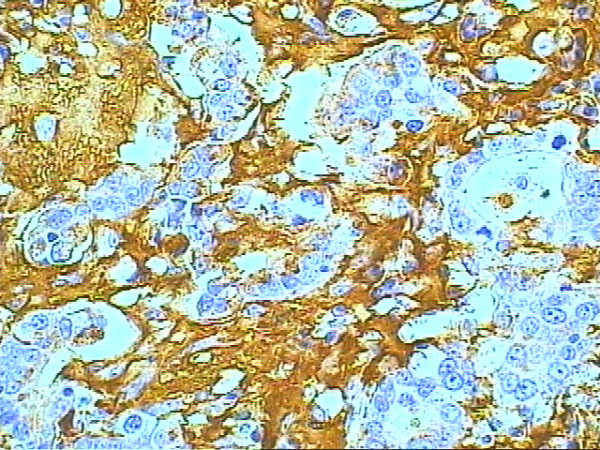
**MUC5AC immunoexpression in undifferentiated type adenocarcinoma (gastric type) arising in Barrett's esophagus**. Immunohistochemical staining of MUC5AC for adenocarcinoma (original magnification × 400).

According to the pattern of mucin expression, four tumors were classified as MUC2 positive (Figure 3) indicating an intestinal type of tumor differentiation, while seven were MUC5AC positive tumors (Figure 4), indicating a gastric type of tumor differentiation. Two undifferentiated tumors had no mucin expression and therefore could not be classified.

**Figure 5:**
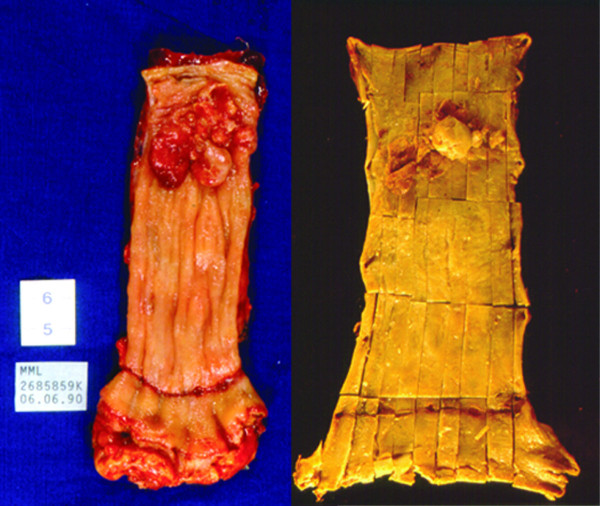
**A protuding proximal Adenocarcinoma over a long Barrett's Esophagus**. Well differentiated adenocarcinoma arising in a 16 cm lenght Barrett's esophagus. The lesion is located 14 cm distant from the gastroesophageal junction.

Figure 5, exemplify an exophytic lesion surrounded by an extensive Barrett's epithelium. Microscopy revealed a well differentiated type tumor. Immunohistochemistry demonstrated a positive MUC2 expression compatible with an intestinal type Adenocarcinoma.

**Figure 6:**
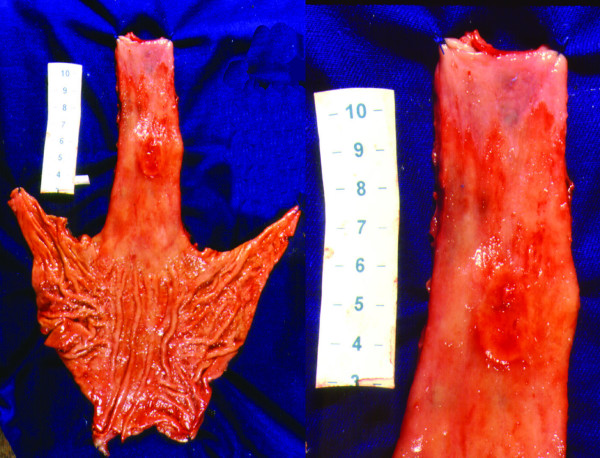
**An infiltrative proximal Adenocarcinoma over a long Barrett's Esophagus**. Undifferentiated adenocarcinoma arising in 10.7 cm lenght Barrett's esophagus, 5.5 cm distant from gastroesophageal junction.

Figure 6, exemplify an ulcerative and depressive lesion surrounded by an extensive Barrett's epithelium. Microscopy revealed an undifferentiated type tumor. Immunohistochemistry showed MUC5AC expression denoting a gastric type Adenocarcinoma.

Table 3 shows the relationship between mucin pattern predominance in the adjacent epithelium compared to the mucin tumour expression.

**Table 3:** Relationship between Mucin pattern predominance in the adjacent epithelium compared to the mucin tumor expression.

	**Adjacent epithelium**	
**Tumor**	**Intestinal**	**Gastric**
**Intestinal**	4	0
**Gastric**	1	6

P = 0, 01 Fisher Exact Test.

## Discussion

The extension of the columnar epithelium in the esophagus is related to the risk of malignant transformation [17,18], and there is an increased odds in BE longer than 4 cm [10,19-21]. Some authors describe the adenocarcinoma in short BE with lower prevalence, since the risk of malignization area (columnar epithelium) is low [7]. In this study, adenocarcinoma developed just in long BE (mean 7.1 cm). This was already observed in our service, when the mean extension of BE who developed the tumor was 9.7 cm [21].

The location of ABE was more frequent next to squamous-columnar transition. Same findings were observed in thirteen patients with early adenocarcinoma [20]. This data suggest that this zone should be specific target during BE follow up, with multiple endoscopic biopsies.

Mucins secreted in the esophagus play an important role in the cytoprotection against reflux of gastric contents [22]. Barrett's mucosa is characterized by a heterogeneous mixture of neutral mucins, sialomucins and sulphomucins [23]. Based on this background information, this study investigated the pattern of expression of MUC2 and MUC5AC mucin gene protein products using immunohistochemistry in patients with adenocarcinoma arising in BE.

MUC2 and MUC5AC belong to a family of mucin genes which encode for peptide tandem repeats [22,24]. Mucin tandem repeats vary in length and sequence, but all characterized to date contain proline, threonine and/or serine residues which are potential glycosylation sites [25], which carry the O-linked oligosaccharides characteristic for these high molecular weight glycoproteins. These mucins are secreted and form extracelular gels [24].

MUC2 encodes a prototype secretory mucin which is present in the human intestine, mostly in goblet cells [26]. The glycopeptide in MUC2 is rich in cysteine residues with disulphide bonds. This results in polymerization and contributes to the intrinsic viscosity and gel-forming properties required for mucosal surface protection [27]. MUC2 immunoexpression in Barrett's metaplasia was restricted to goblet cells, a pattern specific to normal rat and human colonic epithelium [28,29], implying that the mucin in goblet cells of Barrett's metaplasia is similar if not identical to the native intestinal mucosa. Several authors have comparable results [22,30]. The presence of MUC2 in Barrett's metaplasia (goblet cells) is a feature of cellular differentiation because secretory mucins are normally produced by highly differentiated cells [31]. Warson *et al*, 2002, demonstrated that there is an association between MUC2 expression and intestinal metaplasia. Interesting, these authors also found an association between sulphomucin-producing cells and MUC5AC expression [32].

MUC5AC was extensively immunoexpressed in the columnar cells, localizing to the surface epithelium and extending to a variable degree into the glandular structures in BE, and was more commonly seen than MUC2.

In this investigation BE epithelium showed a mucin pattern similar to human stomach epithelium, in which the expression of these MUCs has been demonstrated previously [15,16]. Thus, our finds have been corroborated by others authors.

The metaplastic epithelium may reflect an adaptative response to new luminal environment [14]. The esophagus has been shown to increase secretion of mucins from the submucosal glands in response to stimulation by gastric acid, depending upon the reflux esophagitis [33]. Each region of the gastrointestinal tract has characteristic functional requirements and the properties of the mucus produced at each site are adapted to cope with these functions [34]. Jankowski suggests that incomplete intestinal type metaplasia may be a response to reflux of gastroduodenal contents and in particular bile acids [17]. Arul *et al*. would support a theory as Barrett's epithelium produces both MUC5AC and MUC6 associated with protection from gastric acid and MUC2 and MUC3 associated with protection from bile [14].

Some authors suggested that mucin histochemistry could be used to establish if a pattern of mucin staining in Barrett's esophagus may be associated with a greater risk of progression to adenocarcinoma [35]. Three dyes, alciun blue, high-iron diamine and periodic acid-Schiff reagent are used to histochemically distinghish the mucins produced. These dyes are specific for carbohydrates and their modifications, but do not reveal the underlying molecular identity of the mucins expressed. Expression of sulphomucin has been associated with an increased malignant potencial [35,36]. However, Rothery found that 74% of biopsies of Barrett's esophagus had evidence of sulphomucin and concluded that detection did not help to identify those at risk of progression to adenocarcinoma [4].

NAKAMURA *et al*. performed detailed study of gastric mucosa microcarcinomas, and described the gastric adenocarcinoma histogenesis. They examined stomachs resected for nonmalignant diseases and identified tumor less than 2 mm and between 2 and 5 mm. The results confirmed that mucocelular adenocarcinoma developed from own gastric mucosa, and tubular adenocarcinoma, from atrophic mucosa with IM. After, when he studied tumor greater than 6 mm, he could observe the same relation of the tumor with the adjacent columnar epithelium. With statistical analysis he proved that gastric or undifferentiated adenocarcinoma were related to gastric mucosa (with pyloric or fundic glands), and the intestinal pattern or differentiated adenocarcinoma, with the presence of IM [37].

In this study, the pattern of mucin expression revealed a specialized type epithelium adjacent to the tumors. There was an association between the predominance of mucin expressed in the adjacent epithelium and the pattern of mucin expression in the tumors, may indicating the route of carcinogenesis.

This histogenesis description may be utilized in BE, in order to clarify the presence of gastric mucin type expressed at seven of the ABE in this investigation. So, an area with gastric metaplasia within the specialized Barrett's epithelium could originate an expansion clone capable of initiate the carcinogenesis cascade, developping an undifferentiated adenocarcinoma, that express MUC5AC. BE is a columnar epithelium that can be modified as the gastric mucosa does, and may originate any type of adenocarcinoma.

## Conclusion

Currently, histopathologic aspects still remain the best biologic markers for the BE follow up with the aim of early ABE diagnosis. The location of the adenocarcinoma next to the squamous columnar transition point to the most important zone that should be searched for early adenocarcinona during endoscopic examination; and the higher risk of adenocarcinoma development in long BE, can be used like a red flag for follow up in this patients. Thus, the follow up in long (over 3 cm) BE is relevant, and should be performed in all patients, independently of the type of columnar epithelium found at the endoscopic biopsy.

Therefore, Barrett's esophagus adenocarcinoma shows either gastric or intestinal type pattern of mucins expression. According to the mucins, the two types of tumors developed in Barrett's esophagus may reflect the original cell type involved in malignant transformation.

## Abbreviations

Dist. Tu-GEJ: Distance from tumors (Adenocarcinoma) distal margin to the gastroesophageal junction; Prox. Tu-Tepit: Distance from the turmors (Adenocarcinoma) proximal margin to the epithelium (columnar-squamous) transition; BE: Barrett's Esophagus; ABE: Adenocarcinoma developed in Barrett's Esophagus; HE: hematoxyline-eosine; IM: Intestinal Metaplasia; GEJ: Gastroesophageal junction.

## Competing interests

The authors declare that they have no competing interests.

## Authors' contributions

SS participated in the sequence alignment and drafted the manuscript, design of the study, coordinating data collection, supervision. IC conceived of the study, and participated in its design and coordination, department head. URJ was involved in rewriting, performed the statistical analysis, carried out the immunoassays. KI, REI and CEPC were the pathologists and involved in laboratory investigation. AVSR was involved in collecting data, laboratory investigation, carried out the immunoassays. All authors read and approved the final manuscript.

## References

[B1] Morales TG, Sampliner RE, Bhattacharyya A (1997). Intestinal metaplasia of the gastric cardia. Am J Gastroenterol.

[B2] Spechler SJ, Zeroogian JM, Antonioli DA, Wang HH, Goyal RK (1994). Prevalence of metaplasia at the gastro-esophageal junction. Lancet.

[B3] Thompson JJ, Zinsser KR, Enterline HT (1983). Barrett's metaplasia and adenocarcinoma of the esophagus and gastroesophageal junction. Hum Pathol.

[B4] Rothery GA, Patterson JE, Stoddard CJ, Day DW (1986). Histological and histochemical changes in the columnar lined (Barrett's) oesophagus. Gut.

[B5] Appelman HD, Giuli R, Tytgat GNJ, DeMeester TR, Galmiche JP (1994). Is the presence of specialized epithelium necessary for the diagnosis of Barrett's esophagus?. OESO – The esophageal mucosa.

[B6] Reid BJ (1991). Barrett's esophagus and esophageal adenocarcinoma. Gastroenterol Clin North Am.

[B7] Schnell TG, Sontag SJ, Chejfec G (1992). Adenocarcinomas arising in tongues or short segments of Barrett's esophagus. Dig Dis Sci.

[B8] DeMeester SR, DeMeester TR (2000). Columnar mucosa and intestinal metaplsia of the esophagus – Fifty years of controversy. Ann Surg.

[B9] Sampliner RE (1998). Practice guidelines on the diagnosis, surveillance, and therapy of Barrett's esophagus. The Practice Parameters Committee of American College of Gastroenterology. Am J Gastroenterol.

[B10] Saubier EC, Gouillat C, Samaniego C, Guillaud M, Moulinier B (1985). Adenocarcinoma in columnar-lined Barrett's esophagus. Analysis of 13 esophagectomies. Am J Surg.

[B11] Paraf F, Flejou JF, Potet F, Molas G, Fekete F (1992). Esophageal squamous carcinoma in five patients with Barrett's esophagus. Am J Gastroenterol.

[B12] Paraf F, Fléjou JF, Pignon JP, Fekete F, Potet F (1995). Surgical pathology of adenocarcinoma arising in Barrett's esophagus. Analysis of 67 cases. Am J Surg Pathol.

[B13] Audie JP, Janin A, Porchet N, Copin MC, Gosselin B, Aubert JP (1993). Expression of human mucin genes in respiratory, digestive, and reproductive tracts ascertained by *in situ *hybridisation. J Histochem Cytochem.

[B14] Arul GS, Moorghen M, Myerscough N, Alderson DA, Spicer RD, Corfield AP (2000). Mucin gene expression in Barrett's oesophagus: an in situ hybridisation and immunohistochemical study. Gut.

[B15] Reis CA, David L, Correa P, Correa F, de Bolos C, Garcia E, Mandel U, Clausen H, Sobrinho-Simões M (1999). Intestinal metaplasia of human stomach displays distinct patterns of mucin (MUC1, MUC2, MUC5AC, and MUC6) expression. Cancer Research.

[B16] Ho SB, Shekels LL, Toribara NW, Kim YS, Lyftogt C, Cherwitz DL, Niehans GA (1995). Mucin gene expression in normal, preneopplastic and neoplastic human gastric epithelium. Cancer Research.

[B17] Jankowski JA, Wright NA, Meltzer SJ, Triadafilopoulos G, Geboes K, Casson AG, Kerr D, Young LS (1999). Molecular evolution of the metaplasia-dysplasia-adenocarcinoma sequence in the esophagus. Am J Pathol.

[B18] Reid BJ, Rubin LE (1985). When is the columnar lined esophagus premalignant?. Gastroenterology.

[B19] Cameron AJ, Lomboy CT, Pera M, Carpenter HA (1995). Adenocarcinoma of the esophagogastric junction and Barrett's esophagus. Gastroenterology.

[B20] Nishimaki T, Holsher AH, Schuler M, Bollschweiler E, Becker K, Siewet JR (1991). Histopathologic characteristics of early adenocarcinoma in Barrett's esophagus. Cancer.

[B21] Szachnowicz S, Cecconello I, Iriya K, Marson AG, Takeda FR, Gama-Rodrigues JJ (2005). Origin of adenocarcinoma in Barrett's esophagus: p53 and Ki67 expression and histopathologic background. Clinics.

[B22] Chinyama CN, Marshall RE, Owen WJ, Mason RC, Kothari D, Wilkinson ML, Sanderson JD (1999). Exprerssion of MUC1 and MUC2 mucin gene products in Barrett's metaplasia, dysplasia and adenocarcinoma: an immunopathological study with clinical correlation. Histopathology.

[B23] Lee RG (1984). Mucins in Barrett's esophagus: a histochemical study. Am J Clin Pathol.

[B24] Gum JR (1995). Human mucin glycoproteins: varied structure predict diverse properties and specific functions. Biochem Soc Trans.

[B25] Gum JR (1992). Mucins genes and the proteins they encode: structure, diversity, and regulation. Am J Respir Cell Mol Biol.

[B26] Gum JR, Byrd JC, Hicks JW, Toribara NW, Lamport DT, Kim YS (1989). Molecular cloning of humanintestinal mucin cDNAs. Sequence analysisand evidence for genetic polymorphism. J Biol Chem.

[B27] Gum JR, Hicks JW, Toribara NW, Siddiki B, Kim YS (1994). Molecular cloning of human intestinal mucin (MUC2) cDNA. Identification of the amino terminus and overall sequence similary to prepro-von Willebrand factor. J Biol Chem.

[B28] Tytgat KM, Bovelander FJ, Opdam FJ, Einerhand AW, Buller HA, Dekker J (1995). Biosynthesis of rat MUC2 in colon and its analogy with human MUC2. Biochem J.

[B29] Chang SK, Dohrman AF, Basbaum CB, Ho SB, Tsuda T, Toribara NW, Gum JR, Kim YS (1994). Localization of mucin (MUC2 and MUC3) messenger and peptide expression in human normal intestine and colon cancer. Gastroenterology.

[B30] Ho SB, Niehans GA, Lyftogt C, Yan PS, Cherwitz DL, Gum ET, Dahiya R, Kim YS (1993). Heterogeneity of mucin gene expression in normal and neoplastic tissues. Cancer Research.

[B31] Strous GJ, Dekker J (1992). Mucin-type glicoproteins. Crit Rev Biochem Mol Biol.

[B32] Warson C, Bovenkamp JH Van De, Korteland-Van Male AM, Buller HA, Einerhand AW, Ectors NL, Dekker J (2002). Barrett's esophagus is characterized by expression of gastric-type mucins (MUC5AC, MUC6) and TFF peptides (TFF1 and TFF2), but the risk of carcinoma development may be indicated by the intestinal-type mucin, MUC2. Hum Pathol.

[B33] Namiot Z, Sarosiek J, Rourk RM, Hetzel DP, McCallum RW (1994). Human esophageal secretion: mucosal response to luminal acid and pepsin. Gastroenterology.

[B34] Corfield AP, Myerscough N, Gough M, Brockhausen I, Schauer R, Paraskeva C (1995). Glycosilation patterns of mucins in colonic disease. Biochem Soc Trans.

[B35] Jass JR, Filipe MI (1981). The mucin profiles of normal gastric mucosa, intestinal metaplasia and its variants and gastric carcinoma. Histochem J.

[B36] Duchatelle V, Potet F, Bara J, Ma J, Goldfain D (1989). Mucin immunohistochemistry of the columnar epithelium of the oesophagus (Barrett's oesophagus). Virchows Archiv A Pathol Anat.

[B37] Nakamura K, Sugano H, Takagi K (1968). Carcinoma of the stomach in the incipient phase: its histogenesis and histological appearences. Gann.

